# Comparison of Scapulohumeral rhythm in individuals with and without Forward Shoulder Posture

**DOI:** 10.12688/f1000research.158183.1

**Published:** 2024-11-25

**Authors:** Ganesh Balthillaya M, Shalini H, Indu Reddy, Kiransha R. Velingkar, Eliseus Chettiar, Divya S, Bhamini Krishna Rao, Shyamasunder Bhat N

**Affiliations:** 1 Department of Physiotherapy, Manipal College of Health Professions, Manipal, Manipal Academy of Higher Education, Manipal, Karnataka, 576104, India; 2 Dept. of Orthopedics, Kasturba Medical College, Manipal, Manipal Academy of Higher Education, Manipal, Karnataka, 576104, India

**Keywords:** Round back posture, Scapulothoracic joint, Shoulder abduction

## Abstract

**Background:**

Forward Shoulder Posture (FSP) is one of the most common postural deviations of the upper back, which may affect the scapulohumeral rhythm (SHR). This study aimed to investigate changes in scapulohumeral rhythm during scapular plane shoulder abduction between individuals with and without FSP.

**Methods:**

A cross sectional study with two group comparison. Young adults with FSP (N=39) with a forward shoulder angle ≥ 52°and without FSP(N=40) with a forward shoulder angle < 52° were recruited for the study. Scapular upward rotation was measured with the help of two inclinometers during scapular plane shoulder abduction, at resting shoulder abduction, at 30°, 60°, 90°, 120°, 150°, and at full range of shoulder abduction, and SHR was calculated. The scapulohumeral rhythm was compared between the two groups using an independent *t* test.

**Results:**

Data analysis showed a statistically significant difference in scapulohumeral rhythm at 30°(p=0.004), 60°(p=0.013), and 90°(p=0.009) shoulder abduction between the two groups. There were no significant differences in the other ranges of shoulder abduction between the two groups.

**Conclusion:**

Forward shoulder posture alters the scapulohumeral rhythm between 30 ^°^ and 90°shoulder abduction. Clinicians, trainers, and sports therapists should be aware of this change to intervene at an early stage and prevent the development of pathological conditions around the shoulder.

## Introduction

The scapular position and motion are considered the most important factors contributing to shoulder function. Abnormal scapulothoracic (ST) joint mechanics have been found to be associated with the development of pathological conditions of the shoulder, such as joint instability, impingement syndrome, rotator cuff tendinitis, and other overuse sports injuries of the shoulder. ^1, 2^ Overhead activities require coordinated and balanced movement of the humerus and scapula, scapulohumeral, and scapulothoracic articulations. ^3, 4^ Alterations in the coordination of movement between the scapulothoracic and scapulohumeral articulations affect the scapulohumeral rhythm. ^3– 5^

Forward Shoulder Posture (FSP) is one of the most common abnormal upper back postures. FSP is characterized by protraction of the acromion in front of the line of gravity (LOG), downward rotation, and anterior tilt of the scapula. ^6^ Rounded shoulder posture (RSP) also deviates from the upper back, in which the shoulder is placed anterior to the plumb line in the coronal plane. Several methods have been proposed to measure the FSP. ^7– 9^ Thigpen et al. recommended measuring the forward shoulder angle (FSA) for the diagnosis of forward shoulder posture or round shoulder posture (RSP). FSA is the “angle between vertical line that cross C7 spinous process and the line that pass through the C7 spinous process and acromion”. ^9^ The angle ≥52° was considered as FSP. ^9, 10^ Change in scapular position can cause muscle imbalance, alteration in scapulohumeral rhythm and finally may lead to impingement syndrome, shoulder pain and instability. ^1, 10^

The “scapulohumeral rhythm is the balanced and coordinated movement between the scapula and the humerus. The normal scapulohumeral rhythm and the contribution of scapular motion and humeral motion vary in different ranges of the shoulder complex. ^3^ Coordinated and coupled movement of the scapula and humerus is very important to prevent movement impairments and pathological changes around the shoulder complex. ^11^ Forward head posture and other postural deviations of the upper back and scapula are considered to contribute to changes in scapular motion and scapulohumeral rhythm. ^9^ Alterations in scapular kinematics and scapulohumeral rhythm have also been reported in painful conditions of the shoulder. ^9^ However, studies have not shown a clear relationship between the altered scapulohumeral rhythm and shoulder pain. The presence of pain during testing procedures is a major limitation in these studies, as it makes it difficult to determine the cause for alteration in scapulohumeral rhythm. ^9^ We hypotheses that altered scapulohumeral rhythm would be present between individuals with and without FSP. The identification of altered scapulohumeral rhythm in FSP may provide a theoretical basis for the development of musculoskeletal conditions around the shoulder in FSP. The purpose of this study was to compare scapulohumeral rhythm in asymptomatic individuals with and without FSP and to determine whether there is any difference in scapulohumeral rhythm.

## Methods

Ethical considerations: Study protocol was approved by Ethics Committee of Kasturba Medical College and Kasturba Hospital Manipal (IEC 909-2018, dated 12/12/2018). Written informed consent was obtained by all the participants. All procedures were conducted in accordance with the 1964 Helsinki Declaration and its later amendments.

**Participants:** This was a cross-sectional study with two group comparisons. The study was conducted in department of physiotherapy, Kasturbha hospital manipal, during the period of 12 ^th^ December 2018 to 12 ^th^ March 2019. Seventy-nine young healthy individuals participated in the study through purposive sampling. Sample size was calculated using G-Power software, on the basis of effect size 0.75 (determined by a pilot study), an alpha value 0.05 and desired power of 90%. Individuals age group–20-40 years and with normal shoulder range of motion included in the study. Participants with a forward shoulder angle ≥ 52° on the dominant side were included in the Forward Shoulder Posture (FSP) group (N=39) and participants with a forward shoulder angle < 52° on the dominant side were included in the without Forward Shoulder Posture (FSP) group (N=40). Participants with a history of surgery or traumatic injuries in the upper quarter, pain in the upper quarter within six months of testing, and neuromuscular or musculoskeletal conditions affecting the upper quarter were excluded from the study. Hundred and twelve participants screened for study thirty-three participants excluded from the study as they did not meet the criteria.

### Procedure

**Forward Shoulder Posture assessment:** FSP was determined by measuring the forward shoulder angle (FSA) according to the technique suggested by Thigpen et al. ^9^ FSA was measured in the screening phase by the photogrammetric method. A digital camera was placed four feet in height and seven feet away from the participant. Participants were asked to stand and look forward while maintaining a natural posture. To facilitate the natural posture, participants were asked to move their arms up and down above the head, move their head front and back, and take deep breath three times. The posterolateral part of the acromion process and C7 spinous process were palpated, markers were placed, and photographs were taken from the lateral view. The images were then transferred to a personal computer. The angle formed by the line passing through the C7 spinous process and acromion with a vertical line at the C7 spinous process was measured using an MB-ruler (free software to measure angle and distance) as the FSA. FSA was measured for the dominant and non-dominant sides, and participants with FSA ≥ 52° on the dominant side were considered the Forward Shoulder Posture (FSP) group. Initial screening, measurement of forward shoulder angle, and group allocation were performed by tester 1.

**Scapulohumeral rhythm (SHR) assessment**: The SHR was calculated by measuring scapular upward rotation according to the procedure recommended by Watson et al. ^12^ Two inclinometers were used to measure scapulohumeral rhythm. One inclinometer was used to measure shoulder elevation, and the other was used to measure upward scapular rotation. Measurement of scapulohumeral rhythm was performed during shoulder abduction with elbow full extension, wrist in neutral position, and thumb leading in the scapular plane in relaxed barefoot standing. The first inclinometer was Velcro taped to the humerus just below the deltoid insertion. The dial of the inclinometer rotated such that the dial reading 0° corresponded to the vertical direction. The scapular upward rotation was measured using a second inclinometer by manually aligning the base of the inclinometer along the spine of the scapula during shoulder abduction. Participants were asked to move the shoulder actively from the resting position to 30°, from rest to 60°, from rest to 90°, from rest to 120°, from rest to 150°, and from rest to full range of shoulder abduction, and to hold each position for measurement. Shoulder abduction was measured by the first inclinometer by tester -2 and scapular upward rotation was recorded by 2 ^nd^ Inclinometer by tester -3. Scapular Upward Rotation (SUR) was recorded at the shoulder at rest, at 30°, 60°, 90°, 120°, and 150°, and at full range of shoulder abduction. We conducted reliability testing of the procedure. The procedure was found to be reliable (ICC=0.91) for the measurement of scapular upward rotation at rest, at 30°, 60°, 90°, 120°, and 150°, and at full range of shoulder abduction. Tester-2 and tester-3 were blinded to the group allocation. SHR was calculated as GH elevation/SUR (GH elevation = total shoulder motion-SUR). SHR was measured for dominant upper extremity.

**Data analysis:** The Statistical analysis was conducted using SPSS software version 16.0. Demographic data were analyzed using descriptive statistics. The level of statistical significance was set at P < 0.05. The normality of the data was assessed using the Kolmogorov-Smirnov test. The data were normally distributed. An independent sample *t* test was used to compare scapulohumeral rhythm between the two groups.

## Results

A total of 79 participants (39 in the FSP group and 40 in the non-FSP group) were included in the study. The demographic characteristics of the participants are shown in Table 1.

**Table 1.** Participant demographics and characteristics.

	Forward shoulder posture group	Normal shoulder posture group
Age	24.67 ± 2.89	25.00 ± 3.06
Gender: Male:Female	14:25	16:24
Height in cms (Mean ± SD)	158.87 ± 4.78	158.80 ± 4.72
Weight in kgs (Mean ± SD)	61.87 ± 07.15	59.02 ± 05.85
Hand Dominance: Right:Left	36:3	38:2
FSA in degrees (Mean ± SD)	57.36 ± 2.76	36.85 ± 6.15* (p < 0.001)
Shoulder abduction angle at Rest (Mean ± SD)	7.28 ± 2.05	7.53 ± 1.75 (p = 0.51)
Shoulder abduction angle at end range (Mean ± SD)	176.18 ± 2.72	175.95 ± 3.64 (p = 0.75)
Scapular upward rotation at resting shoulder abduction (Mean ± SD)	3.49 ± 1.43	3.75 ± 1.51 (p = 0.43)

Upward rotation of the scapula at resting shoulder abduction was compared between the FSP and without FSP groups. There was no significant difference in upward rotation of the scapula in the resting shoulder position between the two groups ( Table 1). The independent sample *t* test revealed a significant difference in SHR at 30°, 60°, and 90° of shoulder abduction between the FSP and without FSP group ( Table 2). The difference in scapulohumeral rhythm was not significant at 120°, 150°, and full range of shoulder abduction.

**Table 2.** Comparison of Scapulohumeral Rhythm between with and without FSP group at different angle of shoulder abduction.

Shoulder abduction angle (°)	Scapulohumeral ratio Mean ± SD		Level of significance
	Forward shoulder posture group (FSP)	Without forward shoulder posture group	
At 30°	8.93 ± 1.18	8.30 ± 0.64	0.004
At 60°	7.36 ± 0.82	6.91 ± 0.75	0.013
At 90°	5.63 ± 0.63	5.28 ± 0.51	0.009
At 120°	4.53 ± 0.45	4.38 ± 0.39	0.134
At 150°	3.87 ± 0.37	3.77 ± 0.30	0.198
At full range	3.01 ± 0.23	3.03 ± 0.17	0.650

## Discussion

The purpose of this study was to compare SHR in people with and without FSP. SHR are a key factor in the development of shoulder impairments. Understanding the relationship between SHR and FSP by comparing SHR between individuals with and without FSP may provide valuable information for planning treatment protocols to prevent shoulder impairments. FSP and other postural deviations of the upper back are common in young adults owing to poor postural habits, including the use of smartphones, computers, or laptops. In the current study, young healthy asymptomatic individuals were selected as participants, as postural deviations of the upper back are common in this group. Participants were grouped into the FSP and without FSP groups according to the FSA on the dominant side. To reduce the influence of hand dominance on scapulohumeral rhythm, the SHR was compared on the dominant side in both groups. To the best of our knowledge, no previous study has compared scapulohumeral rhythm between FSP and without FSP. The results of this study revealed that the SHR was significantly greater at 30°, 60°, and 90° of shoulder abduction in the FSP group than in the non-FSP group. This finding indicates that scapular upward rotation is relatively less during shoulder abduction between 30° and 90° of shoulder abduction in persons with FSP than in those without FSP. In this study, we directly compared the scapulohumeral rhythm during shoulder abduction at different angles between the FSP and without FSP groups. Previous reports have compared scapular kinematics and scapular muscle activities to determine their relationship with head and shoulder posture. ^7, 13, 14^

Study by Thigpen et al. compared the scapular kinematics and muscle activity in forward shoulder head posture and Ideal posture during loaded flexion task and reaching tasks. ^9^ There was increased upward rotation of scapula in forward shoulder head posture group compared with ideal posture group during loaded flexion and reaching tasks. ^9^ However, findings of current study cannot be compared with this study due to methodological differences. Scapular upward rotation was assessed during loaded flexion and reaching tasks and in current study scapular rotation was measured during free abduction of the arm in the scapular plane.

Abnormal alignment or posture such as FSP are potential risk factors for the development of shoulder pathologies. ^7^ Relationship between posture and impairment at the shoulder is theorized, but not supported by evidence. ^9^ Sahrmann has proposed the link between alignment deviations and impairment, “alignment deviations are likely to be linked to movement dysfunction and movement dysfunction subsequently leads to impairment”. ^9^ Finding of current study support the Sahrmans proposal by demonstrating connection between alignment and movement dysfunction. According to previous reports, scapular kinematics and scapular muscle activity are affected by head and shoulder posture during shoulder elevation and overhead activities in asymptomatic individuals. ^7, 12, 13^ The current study also supported the hypothesis that FSP impacts scapular mechanics independent of shoulder pain.

Limitations of the current study: 1) Scapulohumeral rhythm was measured only in the scapular plane, and 2) the influence of neck and thoracic posture on forward shoulder angle and scapulohumeral rhythm were not considered.

Future research should consider the categorization of participants based on neck posture, shoulder posture, and thoracic posture to reveal a better understanding of the pattern of alteration in scapulohumeral rhythm.

## Conclusion

Structural alterations such as the Forward Shoulder Posture (FSP) affect scapular kinematics, at least in some ranges of shoulder abduction, which may be a sign of pathomechanical alteration leading to impairment in later stages. We suggest that clinicians, trainers, and sports therapists should be aware of this change to intervene in the early stages and prevent the development of shoulder impairment.

## Ethical review and patient consent

Ethical considerations: Study protocol was approved by Ethics Committee of Kasturba Medical College and Kasturba Hospital Manipal (IEC 909-2018, dated 12/12/2018). Written informed consent was obtained by all the participants. All procedures were conducted in accordance with the 1964 Helsinki Declaration and its later amendments.

## ORCID IDs

Ganesh Balthillaya M: https://orcid.org/0000-0002-2249-0181

Bhamini Krishna Rao: https://orcid.org/0000-0002-2708-0245

Shyamasunder N. Bhat: https://orcid.org/0000-0001-9545-4838

## Data Availability

The data used for the current manuscript will available under the terms of the Creative Commons Attribution 4.0 International license (CC-BY 4.0) in Figshare: Comparison of scapulohumeral rhythm in individuals with and without forward shoulder posture, https://doi.org/10.6084/m9.figshare.27311667.v2. ^15^
