# Supplementary material for: Comparison of Scapulohumeral rhythm in individuals with and without Forward Shoulder Posture
Source: F1000Res. 2024 Nov 25;13:1421. [Version 1] doi: 10.12688/f1000research.158183.1 (PMC11803387; doi:10.12688/f1000research.158183.1)
Supplement: Peer review history

# Reviewer report: Reviewer response for version 1

**DOI:** 10.5256/f1000research.173742.r353042

**Published:** 2025-01-07

**Authors:** Y. Ravi shankar Reddy

**Affiliations:** 1 Department of Medical Rehabilitation Sciences, College of Applied Medical Sciences, King Khalid University, Abha, Aseer Province, Saudi Arabia

Reviewers Report.

**Abstract**

1. Specify the exact measurement methods for scapular upward rotation and shoulder abduction briefly in the methods section of the abstract.
2. Include confidence intervals alongside p-values for the results to improve statistical clarity.
3. Add one sentence about the clinical relevance of altered scapulohumeral rhythm in FSP to strengthen the conclusion.
4. Clarify the rationale for focusing on the scapular plane in the study.

**Introduction**

1. Explicitly state the hypothesis, including the expected differences in scapulohumeral rhythm between FSP and non-FSP groups.
2. Discuss why the scapular plane was chosen for measuring scapulohumeral rhythm rather than other planes of motion.
3. Elaborate on the significance of studying asymptomatic individuals with FSP to the clinical understanding of musculoskeletal conditions.
4. Add a brief explanation of how altered scapulohumeral rhythm might lead to long-term pathological conditions.
5. Correct the grammatical error in the last paragraph: "hypotheses" should be "hypothesize."

**Methods**

1. Justify the choice of purposive sampling and explain measures taken to minimize selection bias.
2. Provide details on how the inclinometers were calibrated and validated before use.
3. Explain why Thigpen et al.'s method for determining FSP and Watson et al.'s method for scapular upward rotation were modified.
4. Include information on whether data were collected in the frontal or scapular plane to avoid ambiguity.
5. Address how confounding factors such as thoracic spine or neck posture were controlled or accounted for.
6. State whether adjustments for multiple comparisons (e.g., Bonferroni correction) were considered to avoid Type I errors.

**Discussion**

1. Expand on the clinical implications of the findings, such as how physical therapy protocols could be adjusted to address altered SHR in FSP.
2. Discuss the impact of the study’s findings on early detection and intervention strategies for shoulder pathologies.
3. Compare the study’s findings directly with Thigpen et al.’s and Watson et al.’s studies, emphasizing differences in methods and outcomes.
4. Address how measuring scapulohumeral rhythm solely in the scapular plane might limit the generalizability of the results.
5. Elaborate on how the limitations (e.g., no control of thoracic posture) might influence the study’s interpretation and application.

**Conclusion**

1. Summarize specific takeaways for clinicians and sports trainers, such as the importance of monitoring scapulohumeral rhythm between 30° and 90° shoulder abduction.
2. Recommend future research to investigate scapulohumeral rhythm in other planes of motion or with consideration of thoracic and neck posture.

Is the work clearly and accurately presented and does it cite the current literature?

Yes

If applicable, is the statistical analysis and its interpretation appropriate?

Yes

Are all the source data underlying the results available to ensure full reproducibility?

Yes

Is the study design appropriate and is the work technically sound?

Yes

Are the conclusions drawn adequately supported by the results?

Yes

Are sufficient details of methods and analysis provided to allow replication by others?

Yes

Reviewer Expertise:

Physical therapy and Rehabilitation.

I confirm that I have read this submission and believe that I have an appropriate level of expertise to confirm that it is of an acceptable scientific standard, however I have significant reservations, as outlined above.

# Reviewer report: Reviewer response for version 1

**DOI:** 10.5256/f1000research.173742.r344533

**Published:** 2024-12-31

**Authors:** Kalyana Chakravarthy Bairapareddy

**Affiliations:** 1 Department of Physiotherapy, College of Health Sciences, University of Sharjah, Sharjah, Sharjah, United Arab Emirates

The manuscript explores an important topic, addressing the impact of forward shoulder posture on scapulohumeral rhythm. The study design, statistical analysis, and discussion provide valuable insights, but there are areas for improvement to enhance the clarity, rigor, and applicability of the findings.

 Major Comments:

 1. The introduction provides a good background on FSP and SHR, but the hypothesis could be more explicitly stated. Consider clearly defining what the expected differences in SHR between FSP and non-FSP groups are.

 2. Sampling Method: While purposive sampling is mentioned, it would be helpful to clarify why this method was chosen and how potential biases were minimized.

 3.  The use of inclinometers is appropriate, but more detail on calibration and measurement reliability would strengthen the study’s validity.

 4. Control of Confounders: The authors briefly mention controlling for hand dominance. However, the influence of other potential confounders (e.g., thoracic spine posture) could be addressed more thoroughly.

 5. While appropriate statistical tests are used, the results section could benefit from more detailed reporting. Specifically, confidence intervals alongside p-values would provide more context about the precision of the estimates. Additionally, the statistical analysis in the manuscript primarily relies on independent *t*-tests to compare scapulohumeral rhythm (SHR) at various angles of shoulder abduction between Forward Shoulder Posture (FSP) and non-FSP groups. While this approach provides basic comparisons, the study can benefit from more advanced methods that account for the repeated measurements across different angles and improve the robustness of the analysis.

 6. The discussion effectively relates findings to existing literature. However, the clinical implications should be expanded. For example, how should physical therapists adjust interventions based on these findings?

 7. The authors acknowledge limitations but do not discuss how these limitations might influence the interpretation of their results.

 Minor Comments:

 1. The tables provide clear data, but adding graphical representations (e.g., bar charts for SHR differences) could improve reader comprehension.

 2. While citations are extensive and relevant, ensuring consistency in formatting (e.g., spacing, punctuation) would improve professionalism.

Is the work clearly and accurately presented and does it cite the current literature?

Yes

If applicable, is the statistical analysis and its interpretation appropriate?

Partly

Are all the source data underlying the results available to ensure full reproducibility?

Yes

Is the study design appropriate and is the work technically sound?

Partly

Are the conclusions drawn adequately supported by the results?

Yes

Are sufficient details of methods and analysis provided to allow replication by others?

Yes

Reviewer Expertise:

Physiotherapy and Rehabilitation

I confirm that I have read this submission and believe that I have an appropriate level of expertise to confirm that it is of an acceptable scientific standard.

# Reviewer report: Reviewer response for version 1

**DOI:** 10.5256/f1000research.173742.r344532

**Published:** 2024-12-11

**Authors:** Peter Rundquist

**Affiliations:** 1 Concordia University, St. Paul, USA

**Introduction**

 Your last sentence in the first paragraph is a bit strong.  Adding may between articulations and affect would be appropriate.

 You have an open quotation mark in the third paragraph without a closed quotation mark.

 Hypotheses in the third paragraph should be hypothesize.  Additionally, the term significant should be added to both your hypothesis and purpose.

**Methods**

 Your method of determining FSP was slightly different than that proposed by Thigpen et al.  What is your rationale for the difference?  Additionally, how reproducible was the methods you used?  The reproducibility question must be addressed to make the article scientifically sound.

 Your method for evaluating scapular upward rotation utilized different elevation angles than Watson et al used.  What is your rationale for the difference? 

 It is unclear if data was collected in the frontal or scapular plane.

**Results**

 To avoid a type 1 error across so many pairwise comparisons, your significance level should be less than or equal to 0.0083 (0.05/6).  Based on this, your only significant difference was at 30 degrees.  This must be addressed to make the article scientifically sound.

**Discussion**

 Did you collect enough data from left hand dominant participants to discuss differences at least narratively between them and right hand dominant participants?

**Data Table**

 You have too many significant digits in several of your SHR columns.

Is the work clearly and accurately presented and does it cite the current literature?

Partly

If applicable, is the statistical analysis and its interpretation appropriate?

Partly

Are all the source data underlying the results available to ensure full reproducibility?

Yes

Is the study design appropriate and is the work technically sound?

Yes

Are the conclusions drawn adequately supported by the results?

Partly

Are sufficient details of methods and analysis provided to allow replication by others?

Yes

Reviewer Expertise:

Shoulder complex kinematics

I confirm that I have read this submission and believe that I have an appropriate level of expertise to confirm that it is of an acceptable scientific standard, however I have significant reservations, as outlined above.
